# Alcohol Use and Its Associated Factors among Adolescents Aged 15–19 Years at Governmental High Schools of Aksum Town, Tigray, Ethiopia, 2019: A Cross-Sectional Study

**DOI:** 10.1155/2021/5518946

**Published:** 2021-03-20

**Authors:** Enguday Tirfeneh Gebeyehu, Mengesha Srahbzu Biresaw

**Affiliations:** ^1^Department of Psychiatry, Aksum University, College of Health Science and Comprehensive Specialized Hospital, Aksum, Tigray Regional State, Ethiopia; ^2^Department of Psychiatry, University of Gondar, College of Medicine and Health Science, Gondar, Amhara Regional State, Ethiopia

## Abstract

**Introduction:**

The impact of alcohol use among adolescents is multidisciplinary and affects the adolescent's academic performance, precipitates with sexually transmitted infections or psychiatric disorders, and disturbs the social domain of adolescents. Therefore, this study aimed to assess the prevalence and associated factors of alcohol use among adolescents aged 15–19 years at the governmental high schools of Aksum Town, Tigray, Ethiopia, in 2019.

**Methods:**

A facility-based cross-sectional study was conducted from 1 to 30 January 2019 at Aksum town high school. Alcohol use was assessed by asking the question “have you used at least one of the alcoholic beverages in the last three months for nonmedical purposes?” Study participants were selected using a simple random sampling technique. Data were collected with face-to-face interview and were analyzed using the Statistical Package for the Social Sciences version 22. Bivariate and multivariate logistic regressions were used to see the association between alcohol use and associated factors. Adjusted odds ratio at a *p* value < 0.05 with a 95% confidence interval was taken to declare the statistical significance of variables.

**Result:**

About 633 adolescents aged 15–19 years were addressed with a response rate of 99.7%. Prevalence of alcohol use was found to be 39.7% [95% CI (35.7, 43.6)]. Being male [AOR = 1.80; 95% CI (1.24, 2.60)], fathers' educational status 1–8 grades [AOR = 2.98; 95% CI (1.60, 5.53)], fathers' occupation farming [AOR = 4.24; 95% CI (2.038.85)], experienced parental neglect [AOR = 1.75; 95% CI (1.20, 2.55)], strong social support [AOR = 1.79; 95% CI (1.11, 2.87)], and family size of greater than five [AOR = 2.03; 95%CI (1.39, 2.97)] were factors identified to be significantly associated with alcohol use among adolescents aged 15–19 years.

**Conclusion:**

In the current study, the prevalence of alcohol use is found to be high when compared to other populations. A strong association has been found between alcohol use and lower paternal educational status and farming as an occupation of parents. There should be a regular awareness creation program for parents with lower education about the devastating effects of alcohol on adolescents.

## 1. Introduction

According to World Health Organization (WHO), alcohol use is defined as a nonmedicinal consumption of psychoactive substances like beer, wine, and whiskey and other alcoholic beverages that have dependence-producing properties which have been widely used in many cultures for centuries [1]. Psychoactive substance use such as alcohol use is a harmful practice when it surpasses the usual recommended dosage. The long-term practice of such harmful practice converts the need to use it to compulsory because of the strong desire to take the drug despite harmful consequences [2]. Worldwide, data indicated that ethanol use is among the significant risk factors for increased morbidity and death rate as well as social disadvantages [3, 4]. It is responsible for the death of 25 million people each year worldwide [1].

Even though the burden is worldwide, developed countries bear significantly higher figures in mortality rates. The World Health Organization (WHO) report in 2004 for the European region showed that alcohol use is responsible for 11% of male deaths [5]. Alcohol use has been recognized as a significant public health issue and imposed an enormous impact on the economy worldwide. Recently, low- and middle-income countries have also reported that alcohol use is incredibly increasing in their communities, including in school-going adolescents [6–8].

The harmful effect of alcohol overweighs among adolescents as it is a critical age group. The impact of alcohol use among adolescents is multidisciplinary and affects the adolescent's academic performance, precipitates to sexually transmitted infections or psychiatric disorders, and disturbs the social domain of adolescents [9]. Furthermore, alcohol use at an early age is reported as it determines adolescents' overall health, sociocultural relationships, and economical status [10, 11].

Studies conducted in different countries, including developing countries, showed that there is an alarming figure on the magnitude of alcohol use among adolescents. The prevalence of alcohol use was reported to be 39.1% in South Africa among high-school adolescents [12], 15% in Kenya [13], and 57.7% and 19.2% in Ethiopia [14].

### 1.1. Justification of the Study

Even though some studies tried to report the magnitude of alcohol use among adolescents, the attention given to interventional policies is minimal. Besides, no study reported its magnitude among a specified age group of late adolescents (i.e., 15–19 years) and evaluated the association between alcohol use and parental treatment and family size of adolescents aged 15–19 years particularly in developing countries. Besides, the late adolescent age group is a stage of life in which adolescents may experience many stressful situations including academic and family-related stresses; this may in turn lead them to use alcoholic beverages as a treatment to get out of their stress [15, 16]. Unless such an influential public health condition on this significant age group is identified and intervened early, it will impose an enormous negative outcome on the community health at large. We found that depicting the magnitude of alcohol use and its associated factors among adolescents aged 15–19 years is pivotal to come up with an effective intervention. Therefore, this study aimed to investigate the prevalence of alcohol use and its associated factors among adolescents aged 15–19 years in Ethiopia.

## 2. Materials and Methods

### 2.1. Study Design, Period, and Setting

This facility-based cross-sectional study was conducted from 1 to 30 January 2019. Adolescents aged 15–19 years at governmental high schools of Aksum town were the target population. Aksum is located in the Tigray region which is 1024 kilometers far from Addis Ababa. Governmental high schools in Aksum town included in this study were Aksum secondary school, Atse/Kaleb secondary school, and Kedamay Menelik secondary school. A total of 4820 adolescents (2579 in grade nine and 2241 in grade ten) were enrolled as students in accessed high schools.

### 2.2. Sample Size Calculation and Sampling Procedure

The study sample size was calculated by using a single population formula taking the following considerations: 95% confidence interval (CI) and 4% marginal error, the proportion of alcohol used 40.9% from the previous study [17], and a nonresponse rate of 10%. The final sample size was taken to be 639. Equal chance to participate was given for all governmental high schools of Aksum town. The numbers of students from each governmental high school were selected proportionally to their total number of students enrolled. Finally, respondents were selected for the study by a simple random sampling, that is, lottery method.

### 2.3. Data Collection Instruments and Techniques

Face-to-face interview was conducted to collect the required information. Initially, the screening tools and other developed structured questionnaires in English language were translated to Amharic and Tigrigna and back to English by an independent person to check for consistency and understandability of the tool. We appointed six data collectors, and the collection process was supervised by the study investigators. Data collectors were trained for clarity of questionnaires and ethical issues.

Alcohol use among adolescents aged 15–19 years was collected by asking a question “have you used/drunk at least one of the alcoholic beverages (beer, wine, whiskey, Areke, Tela, Tej, etc.) for nonmedical purposes within the last three months?” which was answered by “YES” or “NO.” Depressive symptoms were assessed by using a multipurpose instrument PHQ-9. PHQ-9 score of greater than or equal to 10 has sensitivity and specificity of 88% for major depression [18].

History of experiencing parental neglect among adolescents aged 15–19 years was assessed using a 10-item screening tool, that is, Adverse Childhood Experience Questionnaire (ACEQ). Having experienced at least one of emotional abuse, physical abuse, and medical and educational neglect was considered as having parental neglect [19]. The Oslo-3 Social Support Scale was applied to know the level of social support towards adolescents. The scale divides the social support into three levels: poor social support (3–8), moderate social support (9–11), and strong social support (12–14) (reliability Cronbach's *α* = 0.91) [20].

### 2.4. Operational Definitions

 
**Adolescents:** For this study, a school-attending person specifically within 15–19 years of age.
 
**Alcohol use:** Adolescents who answered YES to a question “have you used/drunk at least one of the alcoholic beverages (beer, wine, whiskey, Areke, Tela, Tej, etc.) for nonmedical purposes within the last three months?” were considered as alcohol users [1, 21].
 
**Depression:** Those who score greater than 5 on the PHQ-9 scale [18].
 
**Parental Neglect:** ACEQ, which is a self-report instrument covering 10 items, to rate the severity of emotional abuse and neglect, physical abuse and neglect, and sexual abuse [18].
 
**Social support:** According to the Oslo-3 Social Support Scale, a score of 3–8 is taken as poor support, 9–11 as moderate support, and 12–14 as strong support [20].

### 2.5. Data Analysis and Interpretation

After the questionnaire was checked for cleanliness, the data were entered into EpiData 3.1 and exported to SPSS 22 statistical software for analysis. Frequencies, means, percentages, and standard deviations were calculated for sociodemographic and other independent factors. A logistic regression analysis model was conducted. The relationship between each independent variable and the outcome variable was checked by bivariate logistic regression. Independent variables with a *p* value less than or equal to 0.2 were selected for multivariable logistic regression analysis. Multicollinearity has been checked and chi-square assumptions have been conducted. The significant association of factors was considered at a *p* value of less than 0.05. The strength of association was evaluated using 95% CI and adjusted odds ratio. The model fitness of multivariate regression has been checked by Hosmer-Lemeshow test (*p* value = 0.057).

## 3. Result

### 3.1. Sociodemographic Characteristics

A total of 633 individuals participated in the study with a response rate of 99.06%. More than half, 54.3% (344), of the study participants were female students. The majority of the participants, 69% (437), were orthodox Christians. Regarding the educational level of students, 52.3% (331) were grade nine students and the rest of them, 47.7% (302), were grade ten students. 67.6% (428) of the study participants were from urban areas as regards their residence (Table 1).

### 3.2. Social Support Related Factors

**Figure 1:**
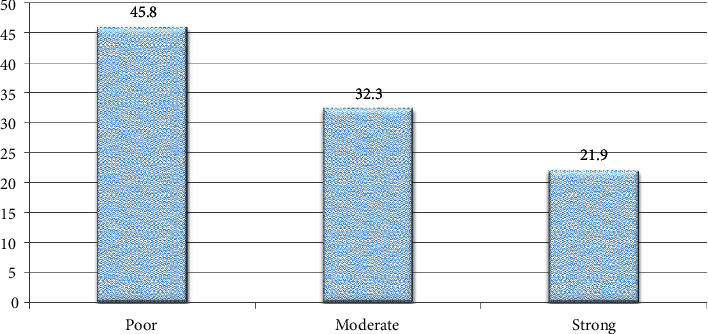
Percentage distribution of level of social support among adolescents aged 15–19 years at governmental high schools of Aksum Town, Tigray, Ethiopia, in 2019 (*n* = 633).

As measured by a three-item Oslo Social Support Scale, 45.8% (290) of adolescents reported that they received poor social support and 32.2% (204) received moderate social support. The rest have been found to be in a strong or good social support level (Figure 1).

### 3.3. Clinical and Behavioral Factors

In addition to current alcohol use by adolescents aged 15–19 years, cigarette smoking and Khat chewing behavior as well as sexual behavior of students were assessed. According to the results, 3.5% (22) and 4.1% (26) of adolescents aged 15–19 years used Khat and tobacco in their lifetime, respectively. The last three-month result indicated that only 15 (2.4%) and 20 (3.2%) used Khat and tobacco. Of the total participants, 28.8% (182) of adolescents aged 15–19 years have suffered from depression. Parental treatments towards adolescents were also screened and the results indicated that about 36% (228) of the students reported that they are neglected either by their parents, caregivers, or guardians. The sexual behavior results showed that 15.6% (99) of adolescent students aged 15–19 years participated in at least one risky sexual behavior.

### 3.4. Prevalence of Alcohol Use and Associated Factors

The overall prevalence of current alcohol use among adolescents aged 15–19 years at high schools in Aksum town was found to be 39.7% (95% CI; 35.7, 43.6). Slightly increased prevalence of alcohol use has been found among grade ten students (74/302) and male students (98/344) when compared to grade nine students (67/331) and female students (43/289), respectively.

The bivariate analysis results showed that variables such as sex of the participant, family size, residence of participants, fathers' educational status, fathers' occupation, and comorbid cigarette smoking lifetime and, within the last three months, level of social support and having experienced parental neglect were found to have a *p* value of less than 0.2. Multicollinearity between independent variables has been checked and chi-square assumptions were done. According to the result, all values of VIF were <1.08 and the correlation coefficient between each variable was less than 0.6. These factors were entered into a multivariate analysis and the results depicted that being male, having a father whose educational status is 1–8 grades, having a father whose occupation is farming, having experienced parental neglect, having poor social support, and increased family size were found to have a statistically significant association with alcohol use among adolescents aged 15–19 years at a *p* value less than 0.05.

The odds of using alcohol among male adolescents aged 15–19 years are 1.80 times higher when compared to those of females [adjusted odds ratio (AOR) = 1.80; 95% CI (1.24, 2.60)]. Adolescents aged 15–19 years whose fathers' educational status is 1–8 grades were 2.98 times more likely to use alcohol than adolescents whose fathers' gaining certificate and above in their education [AOR = 2.98; 95% CI (1.60, 5.53)]. Adolescents aged 15–19 years whose fathers' occupation is farming were 4.24 times more likely to use alcohol than those whose fathers are private employees [AOR = 4.24; 95% CI (2.038.85)].

The probability of using alcohol among adolescents aged 15–19 years who had experienced parental neglect increased by 75% when compared to their counterparts [AOR = 1.75; 95% CI (1.20, 2.55)]. The odds of using alcohol among adolescents aged 15–19 years who are getting strong social support are 1.79 times higher when compared to those who are getting poor social support [AOR = 1.79; 95% CI (1.11, 2.87)]. Adolescents aged 15–19 years who are from a family size of greater than five are 2.03 times more likely to use alcohol when compared to those from the family size of less than or equal to five [AOR = 2.03; 95%CI (1.39, 2.97)] (Table 2).

Goodness of fit as per Hosmer-Lemeshow test (*p*=0.057).

## 4. Discussion

This study showed that the overall prevalence of alcohol use among adolescents aged 15–19 years is 39.7%. This is in line with studies conducted in South Africa (39.1%) [12] and the United States of America (41.8%) [22]. However, this result on the prevalence of alcohol use among adolescents aged 15–19 years was lower than those of two other studies done in Addis Ababa, Ethiopia (45.7%) [23], and in Kisumu, Kenya (51.9%) [24]. The possible explanation for the difference might be the difference in study participants in which a Kenyan study included adults of college students [24] and all adolescents were addressed in a study in Addis Ababa, Ethiopia [18], and patterns of alcohol use assessed in which long-term alcohol use was assessed in a study conducted in Kenya [24] and Addis Ababa, Ethiopia [23].

On the other hand, the result of this study on the prevalence of alcohol use among adolescents aged 15–19 years was found to be higher than those of studies done in the Eastern part of Ethiopia 22.2 and 10.4% for lifetime and past one-month alcohol use [25], another study in Ethiopia 19.4% [14], a study from Kenya 15% and 14% in urban and rural schools, respectively [13], a study from Thailand 14.8% [26] and another from Zimbabwe 15.6% [27]. Such discrepancies might be due to differences in patterns of alcohol use assessment in which one-month alcohol use was assessed in a study from Ethiopia [25] and another from Thailand [26] and the sample size used in which higher sample sizes were used in previous studies [26, 27]. Another possible explanation for the discrepancies might be the difference in the study population in which a separate residency of adolescents was compared in a study from Kenya [13] and participants included in which adolescents aged 12–15 years were studied in Thailand [26].

Besides, the late adolescence age group is a transition from adolescent to early adulthood. The increased chance of late adolescents to experience different stressful situations like increased academic stress, peer influence, family-related stress, and stress related to physical and physiological changes might contribute to the higher prevalence of alcohol use in this age group as they may use alcohol as a stress self-treatment method [28–31].

Regarding the factors associated with alcohol use, our study revealed that being male, having a father learning 1–8 grades, having fathers whose occupation is farming, having experienced parental neglect, having poor social support, and increased family size were significantly associated with alcohol use among adolescents aged 15–19 years at a *p* value less than 0.05.

The odds of using alcohol among male adolescents aged 15–19 years are 1.80 times higher when compared to those of females. This is in line with a study done in the Eastern part of Ethiopia (AOR = 2.09) [25], another study conducted on students of Jigjiga University (AOR = 2.12) [32], and a study done on students of Aksum University (AOR = 2.12) [33]. This association might be because males have been reported to be greatly involved in risky behaviors than females [34, 35]. This might also be due to the cultural influences among males to practice using alcoholic beverages for different purposes [36]. Besides, the difference might be because alcohol is consumed more by males than by females as a means of stress relieving and for recreational purposes [37, 38].

Adolescents aged 15–19 years whose fathers' educational status is 1–8 grades are 2.98 times more likely to use alcohol than adolescents whose fathers' graduated with certificates and above. This result has been supported by a study conducted in Nepal [39]. This association might be because of the lower educational status of parents influencing their ability to address information regarding the harmful effects of using alcohol [40, 41]. Besides, parents of adolescents with lower educational status are more likely to use alcohol, which bears a significant impact on adolescent's alcohol use [42]. Low paternal education may also contribute to their adolescences practicing alcohol use as a means of stress relieving, since they are less likely to learn how to set goals to cope with stress and apply other methods [43].

Adolescents aged 15–19 years whose fathers' occupation is farming were 4.24 times more likely to use alcohol than those whose fathers' are private employees. This has also been supported by a study conducted in Nepal [39]. The possible reason for such association might be because the fact that most farmers have lower educational status [44]. This in turn contributed to continued alcohol drinking despite its harmful effects and later this becomes practiced by their children [45, 46].

The probability of using alcohol among adolescents aged 15–19 years who had experienced parental neglect increased by 75% when compared to their counterparts. This has been supported by different studies [47, 48]. This might be because adolescents experiencing maltreatment and neglect are more likely to develop emotional disorders, which may enforce them to use alcohol as a self-treatment to get out of that emotional feeling tone [18, 46, 49].

The odds of using alcohol among adolescents aged 15–19 years who are getting strong social support are 1.79 times higher when compared to those who are getting poor social support. This study has been supported by a study conducted in India [50]. This might be because there is an increased chance of continuously using alcoholic beverages as far as adolescents get support for alcohol-related expenses [51]. It might also be due to the increased peer pressure to drink alcohol when adolescents have many people around [52].

Adolescents aged 15–19 years who are from a family size of greater than five are 2.03 times more likely to use alcohol when compared to those from a family size of less than or equal to five. This might be because parents may not get a chance to supervise their children as they have an increased number of children [53]. The increased family and social problems when family size increases might also be another contributing factor to the increased prevalence of alcohol use among adolescents [54].

The recruitment of high sample size for the study can be mentioned as the strength of this study. Another quality of this study is that we applied a probability sampling technique during selecting study participants. The study might be affected by recall and social desirability issues as the data collection was interview-administered.

## 5. Conclusions and Recommendations

The prevalence of alcohol use among adolescents aged 15–19 years was found to be high. Being male, 1–8 grades of father's educational status, fathers' occupational farming, having experienced parental neglect, strong social support, and family size of greater than five were factors identified to be significantly associated with alcohol use among adolescents aged 15–19 years.

## Figures and Tables

**Table 1:** Sociodemographic characteristics of adolescents aged 15–19 years at governmental high schools of Aksum town, Tigray, Ethiopia, in 2019 (*n* = 633).

Variables	Category	Frequency	Percentage
Sex	Male	289	45.7
	Female	344	54.3

Educational level	Grade 9	331	52.3
	Grade 10	302	47.7

Religion	Orthodox	437	69
	Muslim	169	26.7
	Protestant	27	4.3

Residence	Urban	428	67.6
	Rural	205	32.4

Fathers' educational status	Illiterate	83	13.1
	1–4th grade	165	26.1
	5–8th grade	144	22.7
	9–12th grade	112	17.7
	Certificate and above	129	20.4

Mothers' educational status	Illiterate	177	28.0
	1–4th grade	138	21.8
	5–8th grade	127	20.1
	9–12th grade	120	19
	Certificate and above	71	11.2

Fathers' occupation	Farmer	222	35.1
	Daily laborer	34	5.4
	Merchant	113	17.9
	Employed	264	41.7

Mothers' occupation	Farmer	178	28.1
	Daily laborer	38	6.0
	Merchant	77	12.2
	Employed	201	31.7
	Housewife	139	22.0

Family size	1–5	377	59.6
	>5	256	40.4

**Table 2:** Bivariate and multivariate logistic analysis of factors associated with alcohol use among adolescents aged 15–19 years at governmental high schools of Aksum town, Tigray, Ethiopia, in 2019 (*n* = 633).

Variables	Category	Alcohol use		COR (95% CI)	AOR (95% CI)	*p* value
		Yes	No			
Sex	Male	155	189	1.65 (1.19, 2.28)	**1.80 (1.24, 2.60)**	**0.002**
	Female	96	193	1	1	

Fathers' occupation	Private employed	27	76	1	1	0.000
	Farmers	114	108	1.63 (.89, 2.97)	**4.24 (2.03, 8.85)**	**0.000**
	Daily labors	8	26	2.85 (1.73, 4.72)	.72 (.27, 1.93)	0.508
	Merchant	48	65	2.95 (1.76, 4.94)	1.65 (.85, 3.19)	0.137
	Government employee	54	107	1.62 (093, 2.83)	1.91 (1.00, 3.64)	0.049

Parental neglect	Yes	107	121	1.60 (1.15, 2.23)	**1.75 (1.20, 2.55)**	**0.004**
	No	144	261	1	1	

Social support	Poor social support	106	184	1	1	0.049
	Moderate social support	84	120	1.22 (.84, 1.75)	1.35 (.89, 2.10)	0.164
	Strong social support	61	78	1.36 (.90, 2.05)	**1.79 (1.11, 2.87)**	**0.016**

Family size	>5	125	131	1.90 (1.37, 2.63)	**2.03 (1.39, 2.97)**	**0.000**
	<−5	126	251	1	1	

Current cigarette smoking	Yes	15	5	4.79 (1.72, 13.36)	4.69 (.89, 24.7)	0.069
	No	236	377	1	1	

Lifetime cigarette	Yes	17	9	3.01 (1.32, 6.87)	1.43 (.36, 5.70)	0.613
	No	234	373	1	1	

Mothers' educational status	Certificate and above	19	52	1	1	0.002
	Illiterate	59	118	1.37 (.74, 2.52)	.76 (.33, 1.75)	0.511
	1–4 grades	71	67	2.90 (1.55, 5.41)	1.69 (.73, 3.93)	0.221
	5–8 grades	57	70	2.23 (1.19, 4.19)	1.91 (.87, 4.20)	0.106
	9–12 grades	45	75	1.64 (.86, 3.12)	1.96 (.93, 4.14)	0.079

Mothers' occupation	Housewife	55	84	1	1	0.076
	Farmer	81	97	1.28 (.81, 2.00)	.63 (.34, 1.16)	0.140
	Daily laborer	11	27	0.62 (.29, 1.36)	0.47 (.19, 1.15)	0.098
	Merchant	34	43	1.21 (.69, 2.12)	1.29 (.67, 2.50	0.451
	Government employee	42	65	0.99 (.59, 1.65)	1.08 (.55, 2.11)	0.827
	Private employee	28	66	0.65 (.37, 1.13)	0.53 (.28, 1.00)	0.051

Residence	Urban	157	271	0.68 (.49, 0.96)	1.16 (0.72, 1.87)	0.547
	Rural	94	111	1	1	

Fathers' educational status	Certificate and above	32	97	1	1	0.002
	Illiterate	29	54	1.63 (.89, 2.97)	1.60 (0.75, 3.39)	0.221
	1–8 grades	151	158	2.90 (1.83, 4.58)	**2.98 (1.60, 5.53)**	**0.001**
	9–12 grades	39	73	1.62 (.93, 2.83)	1.54 (.82, 2.91)	0.182
	Constant				0.04	0.000

## Data Availability

The raw data used to support the findings of this study are available from the corresponding author upon request.
